# Bioinformatic and Molecular Analysis of Satellite Repeat Diversity in *Vaccinium* Genomes

**DOI:** 10.3390/genes11050527

**Published:** 2020-05-09

**Authors:** Nusrat Sultana, Gerhard Menzel, Tony Heitkam, Kenji K. Kojima, Weidong Bao, Sedat Serçe

**Affiliations:** 1 Faculty of Life and Earth Sciences, Jagannath University, Dhaka 1100, Bangladesh; 2 Faculty of Biology, Technische Universität Dresden, D-01062 Dresden, Germany; gerhard.menzel@tu-dresden.de (G.M.); tony.heitkam@tu-dresden.de (T.H.); 3 Genetic Information Research Institute, Cupertino, CA 95014, USA; kojima@girinst.org (K.K.K.); weidong@girinst.org (W.B.); 4 Department of Agricultural Genetic Engineering, Ayhan Şahenk Faculty of Agricultural Sciences and Technologies, Niğde Ömer Halisdemir University, 51240 Niğde, Turkey; sedatserce@gmail.com

**Keywords:** *Vaccinium*, tandem repeat, satellite DNA, higher order repeat (HOR), transposable elements

## Abstract

Bioinformatic and molecular characterization of satellite repeats was performed to understand the impact of their diversification on *Vaccinium* genome evolution. Satellite repeat diversity was evaluated in four cultivated and wild species, including the diploid species *Vaccinium myrtillus* and *Vaccinium uliginosum*, as well as the tetraploid species *Vaccinium corymbosum* and *Vaccinium arctostaphylos*. We comparatively characterized six satellite repeat families using in total 76 clones with 180 monomers. We observed that the monomer units of VaccSat1, VaccSat2, VaccSat5, and VaccSat6 showed a higher order repeat (HOR) structure, likely originating from the organization of two adjacent subunits with differing similarity, length and size. Moreover, VaccSat1, VaccSat3, VaccSat6, and VaccSat7 were found to have sequence similarity to parts of transposable elements. We detected satellite-typical tandem organization for VaccSat1 and VaccSat2 in long arrays, while VaccSat5 and VaccSat6 distributed in multiple sites over all chromosomes of tetraploid *V. corymbosum*, presumably in long arrays. In contrast, very short arrays of VaccSat3 and VaccSat7 are dispersedly distributed over all chromosomes in the same species, likely as internal parts of transposable elements. We provide a comprehensive overview on satellite species specificity in *Vaccinium*, which are potentially useful as molecular markers to address the taxonomic complexity of the genus, and provide information for genome studies of this genus.

## 1. Introduction

Satellite repeats are tandemly organized repetitive sequences present in the genome of all eukaryotes [1,2]. While repetitive sequences constitute a major portion of eukaryotic genomes, the repeat composition is highly variable across living organisms. Up to 85% of the genome can be composed of repetitive sequences, with satellite repeats varying from less than 1% to more than 35%, depending on the plant species or family [1,3,4,5]. Satellite DNAs are non-coding DNA motifs, repeated several hundreds to hundred thousand times, and mostly tandemly organized in monomer arrays [6]. Based on the length of the repeated sequence units, satellite DNAs are subdivided into microsatellites, minisatellites, and satellites. In case of microsatellites, the unit size ranges between 2–6 bp, for minisatellites between 10–40 bp, whereas satellite repeat motifs are generally thought to be longer [6,7,8].

Typically, satellite monomers have a specific size range from 150–180 bp to 320–360 bp, presumably to enable the uniform and phased DNA winding around the histone complexes [6]. Functional importance of the satellite repeats is often associated with their location on the chromosome. For example, plant centromeres of many plants are largely composed of satellite DNA, playing an important role in kinetochore assembly, chromosome stability, and segregation [9,10,11,12]. Similar to centromeric satellite repeats, intercalary and subtelomeric satellite repeats have a significant impact on the structure and organization of chromosomes by the formation of heterochromatin and euchromatin [9,13,14,15]. In addition to the structural significance, satellite repeats can also provide promoter elements, transcription start sites, and binding sites for transcription factors [16,17,18,19]. They strongly contribute to small-RNA-mediated DNA methylation, chromatin status, gene regulation, and thus may influence the expression of neighboring genes [20,21,22,23].

Different mechanisms and genomic regions are thought to be responsible for the origin and amplification of tandemly organized satellite repeats [6]. For example, some satellite repeats show significant sequence similarity to the long terminal repeats (LTR) of LTR retrotransposons [24], miniature inverted-repeat transposable elements (MITEs) [25], intergenic spacers of rDNA sequences [26], or SSR sequences [27], indicative of their possible origin from the respective genomic region. The mechanisms responsible for the amplification of tandemly organized repeat sequences are unequal crossing over, strand slippage, rolling circle-based replication of external sequences, and sequence duplications [7,28]. As adjacent satellite DNA monomers are often simultaneously amplified and spread, some satellite DNAs form higher order repeat (HOR) structures. In an HOR, larger repeated units are composed of multiple basic repeated units [6,29].

Evolution and diversification of satellite repeats of an organism are directly related to environmental and developmental progression [30]. Satellite repeats are often homogeneous on the intra-species level, whereas on an inter-species level, they show higher divergence [7,31]. In some cases, however, mixed distributions with intermediate levels of similarity has been observed for example in apes [32], dogroses [33], *Camellia japonica* [4], or quinoa (*Chenopodium quinoa*) [34,35].

*Vaccinium* is an economically important genus belonging to the Ericaceae family. About 450 different species are recorded in this genus, distributed all over the world. *Vaccinium* is a relatively old genus with a complex evolutionary history, and common interspecific hybridizations—auto- and allopolyploidizations [36,37]. *Vaccinium* species have similar basic sets of chromosomes (n = 12), are crossable, prone to formation of unreduced gametes, and show similar habitat preference with acidic soils [38,39]. Therefore, the species of this genus have been categorized in subgenera or sections. Intersectional hybridization is difficult but not impossible [37,38,39]. Some of the most common and economically important sections are *Cynococcus* (blueberry), *Oxycococus* (cranberry and small cranberry), *Myrtillus* (bilberry), *Hemimyrtillus* (whortleberry), *Vitis-idaea* (lingonberry), and *Vaccinium* (bog bilberry) [40]. The small berry fruits produced by these species contain numerous secondary plant metabolites with antioxidant, anticancer, and antidiabetic activities, providing beneficial health effects [41].

Due to overlapping morphologies and extensive hybridization on interspecies and intersectional level, the *Vaccinium* phylogeny is still under debate [37,42,43]. Although numerous attempts have been taken to reveal the phylogenetic species positions based on DNA and RNA based molecular markers, chloroplast and mitochondrial DNA, their phylogeny is still not resolved, due to data unavailability or nature of the technique [44,45]. Further phylogenetic analysis and germplasm characterization remain essential for the improvement of fruit crops within the *Vaccinium* genus [46].

Genome sizes of diploid highbush blueberries (*Vaccinium corymbosum*) range between 500 Mb to 600 Mb depending on the accession [47], and very recently, a tetraploid highbush blueberry reference genome assembly has become available from cultivar “Draper” with a genome size of 1630 Mb [48]. Moreover, it was established that tetraploid highbush blueberry, a likely allopolyploid with chromosome constitution 2n = 4x = 48, originated from the hybridization of two distinct parental species [48]. The putative parental species of tetraploid cultivated blueberry (*Vaccinium* species) are thought to be the wild diploid bushes—namely, Darrow’s blueberry (*Vaccinium darrowii*), blue ridge blueberry (*Vaccinium pallidum*), or small black blueberry (*Vaccinium tenellum*) [38], which needs further evaluation. Regarding the genus, *Vaccinium* genomes are uniformly sized according to the ploidy level, especially considering the diversity of other genera [3,47,49,50]. We have previously shown that the *Vaccinium* genome is rich in different repetitive sequences and are mainly composed of transposable elements, satellite repeats, rRNA arrays, and a significant amount of unknown repeats [51]. Despite recent advancements in *Vaccinium* genome research, diversity of repetitive sequences in different *Vaccinium* species is still not well studied [48,52,53,54,55,56].

Here, we describe the satellite repeat diversity in the genus *Vaccinium* using molecular and bioinformatic approaches. We analyzed six *Vaccinium*-specific satellite DNA families, in particular focusing on their HOR structures and different degrees of homogenization. Assessment of the satellite repeat diversity enabled the differentiation of four economically important *Vaccinium* species in the sections *Cyanococcus* (*V. corymbosum* L. cultivar ‘Jubilee’ and ‘Misty’), *Hemimyrtillus* (*Vaccinium arctostaphylos* L.), *Myrtillus* (*Vaccinium myrtillus* L.), and *Vaccinium* (*Vaccinium uliginosum* L.). These results open the way towards the use of satellite repeats for the identification and characterization of *Vaccinium* germplasm resources.

## 2. Materials and Methods

### 2.1. Plant Material and DNA Extraction

The wild *Vaccinium* species *V. arctostaphylos* L. (elongated and round fruit type), *V. myrtillus* L. and *V. uliginosum* L. were collected from the Kaçkar Mountains, Rize, Turkey, from July to August during the years 2016–2018 (Table 1). The *V. corymbosum* L. cultivars “Jubilee” and “Misty,” as well as the collected wild species, were grown under greenhouse conditions of Niğde Ömer Halisdemir University. Genomic DNA was extracted from young leaf tissue using the DNeasy Plant Maxi kit (Qiagen, Hilden, Germany) according to the manufacturer’s instructions with some modifications (increased material weight, buffer volume, RNase volume and increased incubation time) when necessary. The phylogenetic relationship among the studied plant sample based on publicly available sequence data of chloroplast maturase K gene (matK gene) and nuclear ribosomal internal transcribed spacer gene (nrITS gene) were recalculated and shown in Figure S1.

### 2.2. Bioinformatic Identification of Satellite DNA Families

Consensus monomer sequences of previously reported satellite repeats for *Vaccinium corymbosum* strain W8520 and *Vaccinium macrocarpon* cultivar “Ben Lear” were used as reference sequence [51]. Paired Illumina genome sequence reads of both genotypes were downloaded from NCBI (SRA053499 and SRA161994). The reads were used for clustering with the RepeatExplorer software [57] with pre-treatment and parameters as described in Sultana et al. (2017) [51]. We used Geneious Prime 2019 for bioinformatic sequence characterization [58]. Based on the graphical output of read clusters, *Vaccinium* satellite candidates have been selected as described in Sultana et al. (2017) [51]. The satellite families VaccSat1 and VaccSat4 differ solely by monomer length, not by sequence [51]. Thus, they belong to the same satellite group and we refer to this group henceforth as VaccSat1. Consensus monomer sequences of satellite repeats from *V. corymbosum* strain W8520 and *V. macrocarpon* cultivar ‘Ben Lear’ were deposited in the www.vaccinium.org database (https://www.vaccinium.org/publication_datasets) and are available under the accession number GDV19001.

For each satellite repeat, we used read-based quantification for the calculation of the copy number in the diploid *V. corymbosum* strain W8520 (genome size 470 Mb): We estimated the genome proportion of each satellite repeat from the RepeatExplorer output in megabases, followed by division of the respective monomer length.

### 2.3. Satellite-Specific PCR Amplification in the Vaccinium Genotypes

Repeat-specific PCR primer pairs were designed from the consensus monomer sequences of the six satellite repeats. In order to produce robust consensus sequences, we refined the RepeatExplorer contig sequences by iterative mapping of Illumina reads. The most homogeneous regions in the mapped reads were inspected for primer design. We designed outward facing primers with a high GC content and lengths between 20 bp and 30 bp, resulting in high stringency (Table 2). PCR amplifications were performed with satellite-specific primer pairs in 50 µl reaction volumes with 50 ng genomic DNA, forward and reverse primers 20 pM each, dNTP 10 mM, Dream taq polymerase (2.5 units) and green taq buffer (10×) (Thermo Scientific, Dreieich, Germany). Standard PCR conditions were 94 °C for 3 min, followed by 35 cycles of 94 °C for 30 s, specific annealing temperature for 50 s, extension at 72 °C for 50 s, and a final extension at 72 °C for 5 min. A gradient PCR was performed for each primer pair to determine the appropriate annealing temperature (Tm). Amplified PCR products were separated by 2% agarose gel electrophoresis (in 3–9 V/cm in 1× TAE buffer (40 mM Tris-Acetate, 1 mM EDTA, pH 8.0) for 35 min. Gels were visualized and analyzed under ultraviolet (UV) light after staining with ethidium bromide. Patterns of PCR amplification for each satellite repeats are recorded in Table S1. Only the combination of genomic DNA and primer pairs showing consistent, multimeric PCR amplicons were considered for satellite repeat cloning.

### 2.4. Cloning, Sequencing, and Raw Sequence Analysis of Satellite Repeats

One genotype from each species was selected for cloning. Therefore, we cloned satellite repeats from the four different *Vaccinium* species (*V. corymbosum* cultivar “Jubilee,” *V. arctostaphylos* elongated fruit type, *V. myrtillus*, and *V. uliginosum*) with six different primer pairs. PCR fragments were purified from the agarose gel using the Invisorb Fragment CleanUp Kit, Berlin, Germany, according to the manufacturer’s instruction. Cleaned PCR fragments were ligated into the pGEM-T Easy vector system following the manufacturer’s instruction (pGEM-T Easy vector system, Promega Corporation, Walldorf, Germany). Ligated products were transformed to XL1-Blue electro-competent cells and recombinant plasmids from the grown bacteria were extracted using the Thermo Scientific GeneJET plasmid miniprep kit following the manufacturer’s instruction. Positive clones were screened by colony PCR with both M13 universal primer pairs and satellite-specific primers pairs. Sanger sequencing was performed with an ABI 3730XL sequencer using the service of BM laboratuvar Sistemleri (https://www.bmlabosis.com/) located in Ankara, Turkey. Raw sequences were analyzed through a mapping strategy where multimeric and monomeric cloned sequences were mapped against the NGS reads of each satellite- specific cluster, using Geneious Prime 2019 [58]. We only proceeded with clones whose sequences mapped against the references. Clone identity was calculated based on the pairwise sequence alignment of the clone sequences and the consensus sequences of respective satellite repeats (Table S1). Nucleotide sequences of satellite clones having an insert length of more than 200 bp were deposited in the NCBI GenBank and are available for download with the accession numbers MK578534-MK578554 and MK567935-MK567947.

Satellite clones with insert sizes of less than 200 bp were deposited in the GDV database (https://www.vaccinium.org/publication_datasets) and are available for download under the accession number GDV19001. Satellite clone characteristics from six individual satellite families are summarized in the Table S1.

### 2.5. Analysis of Structural Variability and Diversity of Satellite Repeats

Multimeric and monomeric clone sequences from individual satellite repeats of different *Vaccinium* species were comparatively analyzed, together with the *V. corymbosum* and *V. macrocarpon* consensus sequences [51].

First, multimeric satellite clones were separated into individual monomer sequences and used for multiple sequence alignment by MAFFT [59], operated by Geneious Prime 2019 [58]. The parameters for MAFFT multiple sequence alignment was default and recorded as Algorithm = Auto (selects an appropriate strategy from L-INS-I, FFT-NS-I, and FFT-NS-2 according to data size), scoring matrix = 200PAM/K = 2, Gap open penalty = 1.53, offset value = 0.123. Pairwise distance matrix values from each alignment were exported. To investigate the effect of the primer regions on the overall identity of each satellite repeat, we both included and excluded the primer regions. Phylogenetic trees were constructed for each individual alignment using the FastTree tool with default parameters [60].

For analysis of the subunit structure of the satellite repeats, we used the Tandem Repeats Finder tool [61] as well as EMBOSS 6.5.7 dotmatcher tool, as integrated in Geneious [62]. Different parameters were used for Tandem Repeats Finder to detect the ideal subunit structure, for instance, with the alignment parameters (match, mismatch, indel = 2,7,7; 2,5,7; 2,5,5; 2,3,5), minimum alignment score to report repeats (20–60), and minimum period size (10–300). The parameters for dotplot analysis of the dotmatcher tool in Geneious were window = high sensitivity slow sliding, score matrix = exact, window size = 14 and threshold = 27. For the analysis of structural variability and homogeneity among satellite monomers from different species all-against-all dotplots comparisons of multimeric clones and multimeric reference consensus sequences were performed. Sequences from each satellite group were analyzed separately using the dotplots software FlexiDot [63]. The parameters for all-against-all sequence comparisons were a word size of 10–12 (-k 10–12), allowing 2–3 mismatches (-S 2–3), based on the pattern of diversity of individual satellite repeats. In the all-against-all comparisons, average pairwise identity of all monomers from the satellite repeats clones and reference sequences were printed.

### 2.6. Analysis of Transposable Elements Similar to Satellite Repeats

The genome assembly of *V. corymbosum* (V_corymbosum_Aug_2015) was downloaded from Blueberry Quickload site for Integrated Genome Browser, and the assembly of *V. macrocarpon* cultivar “Ben Lear” (ASM77533v2) was downloaded from the NCBI Genome Assembly. To identify genomic regions with satDNA, we searched the assemblies with VaccSat consensus sequences as queries with Censor (with default parameters) [64]. The hits were clustered by BLASTCLUST (paremeters: 75% identity, 75% length) of the NCBI Blast package [65] to reveal repetitive regions. Consensus sequences were generated from each of the resulting clusters. To characterize the complete repeat unit, we run Censor against the genome with the consensus sequence for each cluster and extracted the genomic coordinates with the flanking sequences. The consensus sequences representative for new transposable element families have been annotated, named according to the universal classification system implemented in Repbase [66], and submitted to Repbase database [67].

### 2.7. Analysis of the Distribution of Satellite Repeats Monomer along the Pseudochromosomes of the Tetraploid V. corymbosum

We have analyzed the distribution along the 48 pseudochromosomes of the tetraploid highbush blueberry genome from the recently published chromosome-scale genome assembly by Colle et al., 2019 [48]. We used the “Annotate from database” tool from Geneious Prime 2019 for this analysis [58]. The assembled genome sequences of the 48 pseudochromosomes of tetraploid highbush blueberry (*V. corymbosum* cultivar “Draper”) genome was downloaded from the GigaScience database (GigaDB) [48]. Consensus monomer sequences of six *Vaccinium* specific satellite repeats (VaccSat1-VaccSat3 and VaccSat5-VaccSat7) from *V. corymbosum* strain “W8520” were used as a reference sequence database. Annotation of each satellite was performed with an 80% similarity level along the length of each satellite repeat monomer and pseudochromosome. After extensive parameter testing, the 80% similarity level was chosen, since *Vaccinium* satellites showed a high sequence divergence and transposable element similarity. After the annotation process, each satellite repeat feature was counted for each pseudomolecule, and charts were prepared from those data.

## 3. Results

### 3.1. Analysis of VaccSat Satellite Repeats by PCR

Using cluster analysis of sequence reads, we previously identified six satellite DNA families from the genome sequence of the *V. corymbosum* strain W8520 and the *V. macrocarpon* cultivar “Ben Lear” [51]. Characteristic features of these six satellite repeat families from *V. corymbosum* named *Vaccinium* satellite DNA (VaccSat), including an estimated copy number, have been summarized in Table 3. As VaccSat1 and VaccSat4 consensus sequences were 82.8% similar [51], they belong to the same satellite group and were analyzed together.

Here, VaccSat-specific primers pairs (Table 2) were designed from these six satellite families and used to analyze their genomic organization in six economically relevant genotypes from four different *Vaccinium* species. The species included *V. corymbosum* (two cultivars), *V. arctostaphylos* (two populations), *V. myrtillus* (one population), and *V. uliginosum* (one population) (Table 1).

**Figure 1:**
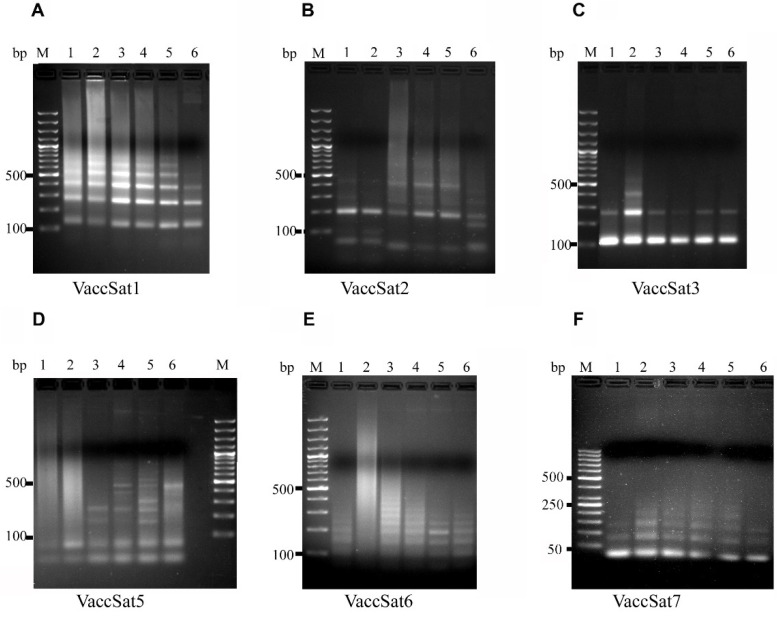
Amplification of *Vaccinium* satellites in different species using satellite-specific primers. (**A**–**C**) VaccSat 1–3, (**D**–**F**) VaccSat 5–7. The plant species used and molecular marker were represented in different lanes and are as follows: Lane 1: *V. corymbosum*, cultivar “Jubilee,” Lane 2: *V. corymbosum* cultivar “Misty,” Lane 3: *V. arctostaphylos* (elongated shaped fruit), Lane 4: *V. arctostaphylos* (round shaped fruit), Lane 5: *V. myrtillus*, Lane 6: *V. uliginosum.* Lane M: 100 bp/50 bp DNA marker.

After separation of the PCR products, the smallest amplicons of all families were conserved in length across the analyzed genotypes, indicating conserved monomer lengths. Nevertheless, the banding patterns varied for different satellite repeats and plant genotypes (Figure 1 and Table S1), and are summarized below for each repeat.

The primer pairs specific for VaccSat1/VaccSat4 (Table 2) produced a clear satellite-typical ladder-like PCR pattern in all genotypes, starting with a monomeric band of 119 bp (Table 2 and Figure 1A). The first expected monomeric band of 119 bp was shorter than the size of the monomer sequences of 146 bp, because of the primer positions along the consensus monomer (Figure S2 and Table 2). All genotypes produced distinguishable amplicons up to tetramers and hexamers, except for *V. uliginosum* (lane 6), where the ladder-like PCR pattern above the trimeric band was less pronounced (Figure 1A and Table S1).

The satellite-typical banding pattern of VaccSat2 showed the expected monomeric sizes of 198 bp according to the primer design on the consensus monomer sequences (Figure S2, Figure 1B, and Table 2). All genotypes produced a similar distinguishable banding pattern up to dimers and trimers. However, the size of the trimer of *V. uliginosum* (lane 6) was about 400 bp and thus was much shorter than the trimeric bands of other genotypes, which have as size of approximately 450 bp. Additional bands not corresponding to the expected multimeric sizes were also amplified in different genotypes (Figure 1B, lanes 1–6). This phenomenon might be related to the fact that the primers may bind twice within each VaccSat2 monomer as depicted in Figure S2. Nonetheless, additional parallel bands of approximately 180 bp were only present in *V. uliginosum* (lane 6), but absent in other species (Figure 1B and Table S1).

VaccSat3-specific primers resulted in clear, monomeric bands of 111 bp in all genotypes studied (Figure 1C and Table 2), corresponding to the expected VaccSat3 repeating unit and primer binding site (Figure S2). However, we observed different amplification of the satellite ladder for different species, detectable as di-, tri-, and tetrameric bands for *V. corymbusum* “Misty” (lane 2), dimers only for *V. arctostaphylos* (lane 4), and dimers and trimers for the remaining species. Moreover, no unspecific, additional banding pattern or primer binding sites were detected in VaccSat3 (Figure S2, Figure 1C, and Table S1).

The VaccSat5 primer pairs produced the envisaged dimeric band (55 bp), clearly amplified in all species investigated (Figure 1D and Table 2). As VaccSat5 monomers are short (approximately 36 bp), the primers were designed in a way that the first expected band represented the dimer of 72 bp (Figure S2). However, the VaccSat5 PCR products were the most diverged satellite amplicons in the *Vaccinium* genotypes studied (Figure 1D and Table S1). For exampleVaccSat5 did not show any ladder-like patterns, but only smears in *V. corymbosum* (lanes 1 and 2) and *V. uliginosum* (lane 6). Nevertheless, ladder-like banding patterns were observed in *V. arctostaphylos* (lane 3 and 4) and *V. myrtillus* (lane 5), superimposed on a light smear (Figure 1D). We amplified bands up to the tetramer and heptamer for two populations of *V. arctostaphylos* (lanes 3 and 4) and a heptamer for *V. myrtillus* (lane 5). Differential amplification and unregular ladder increments throughout the genotypes indicates that VaccSat5 has the tendency toward genotype- and species-specific amplification (Figure 1D and Table S1).

VaccSat6 has a small monomer size of 49 bp. The first amplicon of 54 bp represents the expected band, according to the primer design and short monomer size (Figure S2 and Table 2). This PCR product was present in all species (Figure 1E, lanes 1 and 3–6, and Table S1), except for *V. corymbosum* (lane 2), where only a smear was detected. In addition, VaccSat6 produced smears in all studied genotypes (lanes 1–4 and 6) except for *V. myrtillus* (lane 5), where the bands were clearer and produced a satellite-specific banding pattern (Figure 1E). We amplified a VaccSat6 satellite ladder up to the octamer for *V. arctostaphylos* (lanes 3 and 4), up to the hexamer for *V. myrtillus* (lane 5) and *V. uliginosum* (lane 6), superimposed upon a background smear (Figure 1E and Table S1). The smear may indicate an unspecific primer binding sites and unspecific amplification in the studied genotypes.

We amplified ladder patterns for VaccSat7 in all analyzed species, with a monomeric band corresponding to the expected monomer size (79 bp) and primer design (Figure S2, Figure 1F, and Table 2). The amplified satellite ladder band was up to a dimeric band in *V. corymbosum* cultivar “Jubilee”: (lane 1) and *V. uliginosum* (lane 6). However, amplification up to the tetramers was observed in the remaining genotypes and species (Figure 1F and Table S1).

### 3.2. Satellite repeat Diversity and Organization across Different Vaccinium Species

**Figure 2:**
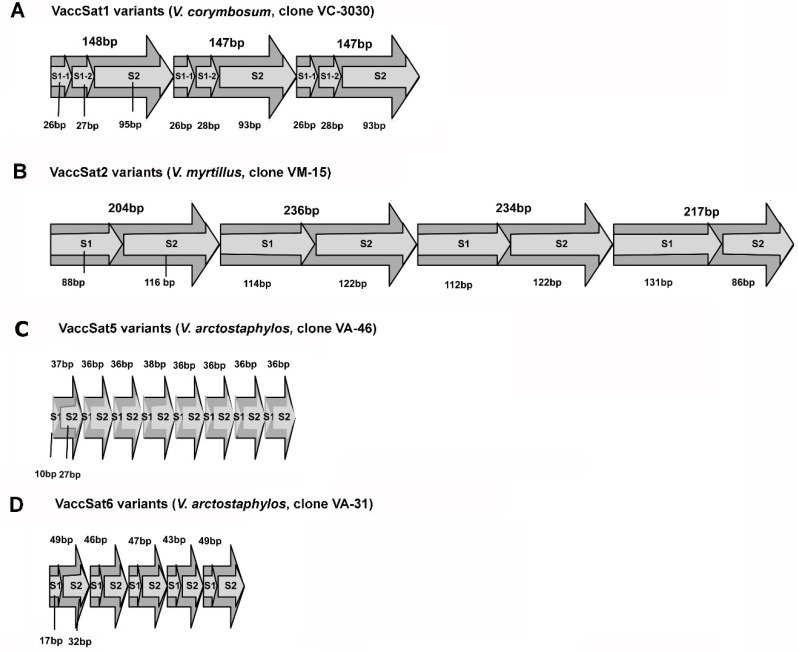
Higher order repeat (HOR) structure of *Vaccinium* satellite repeats. HOR patterns from the longest multimeric clones of different *Vaccinium* species have been selected as representative examples. (**A**) Structure of VaccSat1 from *V. corymbosum*, clone VC-3030; (**B**) structure of VaccSat2 from *V. myrtillus*, clone VM-15; (**C**) structure of VaccSat5 from *V. arctostaphylos* (elongated fruit), clone VA-46; (**D**) structure of VaccSat6 *V. arctostaphylos* (elongated fruit), clone VA-31. Length (bp) of each individual monomer sequences are shown above each monomer sequence. S1 = Subunit1, S1-1 = first variant of Subunit1, S1-2 = second variant of Subunit 1, S2 = Subunit2.

**Figure 3:**
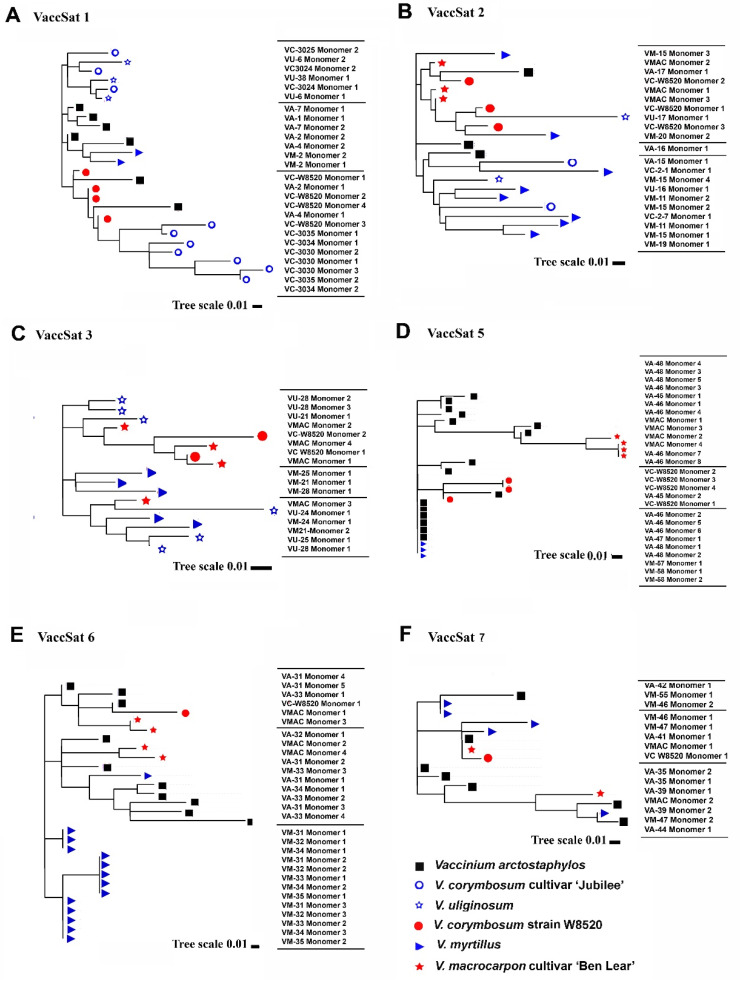
Phylogenetic analysis of satellite monomer sequences from different *Vaccinium* species using FastTree 2.1.5 [55]. (**A**–**C**) VaccSat 1–3; (**D**) VaccSat 5; (**E**) VaccSat 6; and (**F**) VaccSat 7. VA = *V. arctostaphylos*, VC = *V. corymbosum* cultivar “Jubilee,” VC-W8520 = *V. corymbosum* strain “W8520” (reference sequence), VM = *V. myrtillus* and VU = *V. uliginosum*, VMAC = *V. macrocarpon* cultivar “Ben Lear” (reference sequence). Each species is differentiated by each individual symbol.

To enable a sequence comparison across different *Vaccinium* species, we cloned and sequenced the VaccSat PCR products. After sequencing, 180 monomers were extracted from 76 satellite DNA clones from all species. The number of sequenced clones is shown in Table 4; out of those, 103 were of full length. Due to species-specific amplification and diverse sequence lengths, we obtained less cloned sequences for VaccSat5, VaccSat6, and VaccSat7 compared to VaccSat1, VaccSat2 and VaccSat3 (Table 4). Monomers from the reference genomes of the *V. corymbosum* strain W8520 and the *V. macrocarpon* cultivar “Ben Lear,” and the full length monomers from the studied species were analyzed in detail by dotplot analyses, multiple sequence alignments, and their representation as dendrograms to determine their internal subunit structure with conserved and polymorphic sites (Figure 2 and Figure 3, Figures S3–S6, and Table 4 and Table S2).

To investigate the effect of the homogenous primer region on the monomer identity values, we calculated all values with inclusion and exclusion of the primer region (Table S3). In most cases, for VaccSat1, VaccSat2, VaccSat3, and VaccSat6, the identity values were nearly similar. As VaccSat5 consists of 40 bp monomers, completely covered by primers, an exclusion of the primer region has not been possible. For VaccSat7, the primer regions contribute 67 to 90% of the monomers. If we exclude this relatively large region, the average VaccSat7 monomer similarity drops to 68%. Therefore, we conclude that for most of our analyses, the effect of the primer region on the calculated identities is negligible; nevertheless, all values are available in Table S3.

#### 3.2.1. VaccSat1

Multiple sequence alignments of full-length monomer sequences and dotplot analyses of multimeric clones of different *Vaccinium* species revealed that VaccSat1 had variable monomer lengths ranging from 138 to 148 bp and harbored subunits repeated in higher order (Figures S3A and S4A). The longest VaccSat1 arrangement was cloned from *V. corymbosum*, exemplarily showing its S1-1 + S1-2 + S2 HOR structure, with two short, directly repeated subunits S1-1 and S1-2, followed by a longer subunit S2 (Figure 2A). This repeating structure was present in all studied plant species (*V. corymbosum*, *V. arctostaphylos*, *V.myrtillus*, and *V. uliginosum*), but was most prominent in *V. uliginosum* compared to other three species (Table S2 and Figures S3A and S4A). The subunit alignment of S1-1 and S1-2 verified the split into two distinct sequence groups (Figure S5A–C). The pairwise identity between S1-1 and S1-2 ranged between 67–41% (Figure S5F), which signifies their high diversification. While the lengths of S1-1 and S1-2 are consistent in all studied species, ranging between 26–28 bp, S2 lengths are highly variable within and across the species (Figure S4A and Table S2). The S2 length differences were most prominent in *V. arctostaphylos*, ranging between 39 bp and 94 bp. In contrast, the remaining species had more similar S2 lengths varying between 85 bp and 96 bp (Table S2). Presence of this HOR indicates the origin and proliferation of a larger repeat motif from duplication of a shorter repeat. If the HOR structure persists in the population, the smaller repeat motif may accumulate mutations and become unrecognizable in some species (Figure S3A).

To explore VaccSat1 diversity in different *Vaccinium* species, a dendrogram from full-length monomers of different species was prepared (Figure 3A). We found that VaccSat1 monomers diversified in a species-specific manner in *V. arctostaphylos*, *V. corymbosum* “Jubilee,” and *V. corymbosum* “W8520.” This is reflected by pairwise identity values, ranging from 67–97%, indicating variable monomers on the inter- and intra-species level representing the complex sequence organization and structure (Table 4 and Figure 3A).

#### 3.2.2. VaccSat2

To investigate the VaccSat2 subunit structure and monomer length, we generated dotplots from the multimeric clones and multiple sequence alignment of full = length monomer sequence (Figures S3B and S4B). We found that overall monomer length varied from 201 to 240 bp and was made up of two subunits (Figures S3B and S4B). Although subunit organization of VaccSat2 was detected in all species, we observed more homogeneous organization in *V. corymbosum* “Jubilee” and *V. arctostaphylos*, and fragmented organization in *V. myrtillus* and *V. uliginosum* (Table S2 and Figures S3B and S4B). The repeat structure of VaccSat2 was analyzed from the longest satellite clone from *V. myrtillus*, showing a HOR structure with the two highly similar subunits S1 and S2 (Figure 2B and Figure S6A–C). They are marked by subunit-specific polymorphisms and are subsequently grouped in the phylogenetic dendrogram (Figure S6B,C). Sequence polymorphisms and length variations between S1 and S2 (74–34%; Figure S6F) indicate high subunit diversification. Subunit S1 is comparatively longer than S2 in most of the cases and has a consistent length in all species (117–120 bp), except for *V. myrtillus* (88–138 bp) (Figure S4B and Table S2). The length variation of S2 is similar in three wild species (*V. arctostaphylos*, *V. myrtillus*, and *V. uliginosum*) ranging from 79 bp to 120 bp, compared to cultivated *V. corymbosum* “Jubilee” with sequences ranging from 116 bp to 118 bp (Figure S4B and Table S2). Similar to VaccSat1, the presence of HORs in VaccSat2 may indicate the origin of the satellite monomer from duplication of a shorter motif.

For VaccSat2, we observed pairwise identities of 71 to 90% with the consensus monomer across all species (Table 4). Clustering in phylogenetic dendrograms from full-length VaccSat2 monomers verifies the lack of species-specific cluster (Figure 3B).

#### 3.2.3. VaccSat3

For VaccSat3, multiple sequence alignment and dotplots clearly revealed monomers in the length range between 152 bp and 154 bp in different *Vaccinium* species without HOR arrangement (Figures S3C and S4C). Regardless of the species, the VaccSat3 monomers are generally homogeneous on intra- and interspecies level (Figure S4C). However, as all-against-all dotplot comparison showed, some VaccSat3 clones harbor variation in satellite arrangement. For instance, satellite clone VM-24 of *V. macrocarpon* cultivar “Ben Lear” had an inconsistent satellite arrangement (Figure S3C), indicating that homogenization might not yet be completed across the species.

The dendrogram from the extracted monomer sequences for VaccSat3 satellite repeats showed four partially species-specific clusters (Figure 3C). The pairwise sequence identities for VaccSat3 ranged between 65–91% (Table 4) and reflects the sequence variability on the intra- and interspecies level and the clustering in the dendrogram (Figure 3C).

#### 3.2.4. VaccSat5

As VaccSat5 has been amplified differently from different species, multimeric satellite clones were only derived from *V. arctostaphylos* and *V. myrtillus*, but not from *V. corymbosum* (cultivar “Jubilee”) and *V. uliginosum* (Figure 1D and Table 4). VaccSta5 monomer sequences ranged between 36 bp and 38 bp (Figures S3D and S4D). The longest VaccSat5 clone belonged to *V. arctostaphylos* species, serving as a model to illustrate the arrangement of higher order in all species (Figure 2C). The VaccSat5 monomer is constituted of two subunits (S1 + S2) with different lengths (Figure 2C), consistently identified in all investigated species (Figures S3D and S4D). The S1 length of 10 bp was constant in all studied species. However, the S2 length differed between species, with 26 bp in *V. myrtillus* and 26–29 bp in *V. arctostaphylos* (Figure S4D and Table S2).

Dendrograms generated from the full-length monomer sequences of the studied accessions showed that VaccSat5 monomers partially group together in species- and genotype-specific manner (Figure 3D). For instance, there are species-specific clusters belonging to *V. arctostphylos* and *V. myrtillus* as well as to the reference sequences, and another one belonging to *V. macrocarpon* (Figure 3D). The pairwise identities of the cloned monomers to the consensus ranged from 88% to 97% (Table 4), reflected also by the topology of the dendrogram (Figure 3D).

#### 3.2.5. VaccSat6

In case of VaccSat6, due to differential PCR amplification with significant amount of smear in the studied *Vaccinium* genotypes, we obtained multimeric satellite clones from *V. arctostaphylos* and *V. myrtillus*, only (Figure 1E and Table 4). The analyzed VaccSat6 monomers varied between 43 bp and 52 bp, arranged in HOR structures (Figures S3E and S4E). We detected similar S1 + S2 HOR arrangements in all analyzed species, here exemplarily presented for the longest VaccSat6 clone from *V. arctostaphylos* (Figure 2D). S1 had a constant length of 17 bp in both analyzed accessions as well as in the reference sequences (Table S2), whereas S2 had a conserved length of 26 bp in *V. myrtillus* but a variable length in *V. arctostaphylos* ranging between 26 bp and 32 bp (Figure S4E and Table S2).

Dendrograms from monomer sequences of the analyzed *Vaccinium* species revealed species-specific variants for *V. arctostaphylos* and *V. myrtillus* (Figure 3E). The different pairwise identities for each VaccSat6 clone to the consensus sequence (81–90%) are in line with the species-specific clustering of monomer sequences (Table 4 and Figure 3E).

#### 3.2.6. VaccSat7

Like VaccSat5 and VaccSat6, multimers of VaccSat7 were only amplified and cloned from *V. arctostaphylos* and *V. myrtillus*. Monomer lengths of VaccSat7 were variable across species and reference sequences, ranging between 49 bp and 72 bp (Figures S3F and S4F). The satellite monomers showed no arrangement as HOR (Figure S3F). However, multiple sequence alignments of monomers from different species revealed a heterogeneous region, composed of a variable “CAAAAAAA” motif. *V. arctostaphylos* and *V. myrtillus* lack the “CAAAAAAA” motif, but it is present in the *V. macrocarpon* cultivar ‘Ben Lear’ and the *V. corymbosum* strain ‘W8520′ (Figure S4F). A dendrogram from the monomers revealed no species-specificity (Figure 3F). Pairwise identities between the cloned sequences and the consensus monomer sequences ranged from 66% to 78%, reflected by their position in the dendrogram (Table 4 and Figure 3F).

### 3.3. Similarities and Associations of Satellite Repeats with TEs

**Figure 4:**
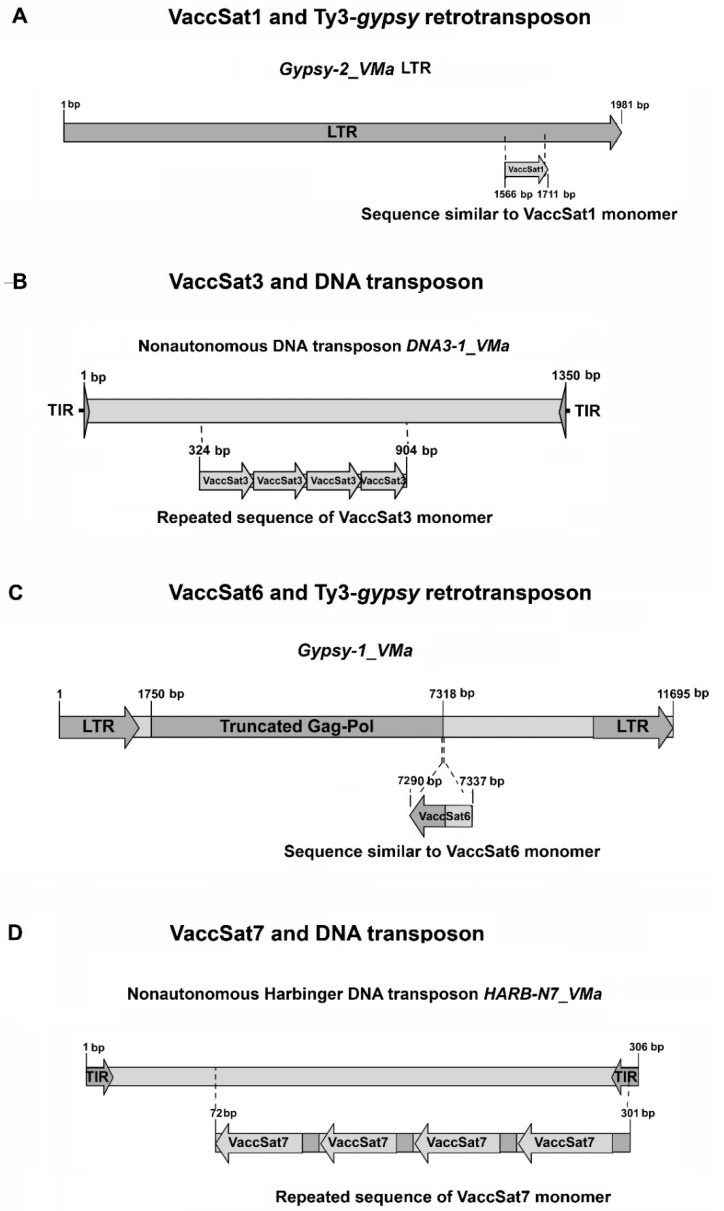
Schematic diagrams of satellite–TE associations in *V. macrocarpon*. (**A**) A VaccSat1-like sequence has been detected in solo-LTRs of the *Gypsy-2_VMa* retrotransposon family. (**B**) Four tandem VaccSat3 units has been identified within *DNA3-1_VMa,* a nonautonomous DNA transposon family. (**C**) A VaccSat6-like sequence has been detected within *Gypsy-1_VMa* retrotransposon family. Darker rectangle shows the truncated Gag-Pol protein coding region. (**D**) Four tandem VaccSat7 units has been detected within *HARB-N7_VMa* nonautonomous *Harbinger*-type DNA transposon family. Here, the rectangles represent transposable elements (TEs) and arrows represents long terminal repeats (LTRs) region or terminal inverted repeats (TIRs) region satDNA monomer.

To test for putative similarities or associations of tandem repeats with TEs, we analyzed the flanking VaccSat regions using the *V. corymbosum* and *V. macrocarpon* genome assemblies. We identified associations between satellite DNAs and TEs for four of the six VaccSat families. Whereas a tandem arrangement of several monomers has been observed inside TEs for VaccSat3 and VaccSat7, satDNA-like sequences in TEs without a tandem organization were detected for VaccSat1 and VaccSat6 (Figure 4 and Figure S7).

Although we did not detect tandemly arranged VaccSat1 monomers in the genome of *V. macrocarpon*, dispersed sequences similar to VaccSat1 are present, corresponding to parts of the LTRs of a Ty3-*gypsy* retrotransposon named *Gypsy-2_VMa* in the Repbase database. A total of four and 15 full-length copies of *Gypsy-2_VMa*-LTR were detected from cranberry and blueberry genome assemblies, respectively. No internal protein coding regions were found for these elements. Therefore, in that context, *Gypsy-2_VMa*-LTR can be categorized as solo-LTR. Nevertheless, in *V. macrocarpon*, close similarity of *Gypsy-2_VMa*-LTR to an *Ogre*-type Ty3-*gypsy* family points to an origin of this solo-LTR from an *Ogre* element. This may further indicate that VaccSat1 originated from a Ty3-*gypsy* LTR in the common ancestor of *V. corymbosum*, *V. arctostaphylos*, *V. myrtillus* and *V. uliginosum*, after the divergence of *V. macrocarpon* (Figure 4A and Figure S7A,B).

In the case of VaccSat3, we observed tandemly repeated VaccSat3-like units in three DNA transposon families, *DNA3-1_VCo* from *V. corymbosum* and *DNA3-1_VMa* as well as *DNA9-20_VMa* from *V. macrocarpon*. The members of the *DNA3-1_VCo* and *DNA3-1_VMa* families are flanked by 3 bp target site duplications (TSDs), the hallmarks of transposon insertions. The VaccSat3 monomers show 92–97% sequence identity to the VaccSat3 consensus sequences in the above mentioned two DNA transposons families (Figure 4B). Similarly, *DNA9-20_VMa*, which is flanked by 9 bp TSDs, also contains VaccSat3-like sequences in tandem with an identity of 80–89% to the VaccSat3 consensus sequence. Although these three families are the only transposon families with VaccSat3 units in tandem, other transposon families contain monomeric sequences similar to a part of VaccSat3 repeat unit which are more diversified and not arranged in tandem. Sequence identity of VaccSat3 units ranged between 72–95% to the consensus monomer sequences in these families. For instance, non-autonomous DNA transposons of the *Mariner*-type often contain sequences similar to the 5′ part of VaccSat3. Besides, one *Helitron* family contains a sequence similar to VaccSat3, too.

In addition, *DNA8-5_VMa* and *DNA8-7_VMa*, flanked by 8 bp TSDs, contain sequences similar to a part of VaccSat3 (Figure 4B and Figure S7C). These transposon families are not related to each other, thus indicating independent capture of VaccSat3-like sequences by different TE types.

Sequences similar to VaccSat6 were found in *V. macrocarpon*, both with and without tandem arrangement. Tandemly arranged sequences are composed of complete VaccSat6-like units without TE association, whereas individual VaccSat6-related units have also been detected within *Ogre*-like Ty3-*gypsy* retrotransposons, named as *Gypsy-1_VMa* in the Repbase database. This may indicate that VaccSat6 was derived from *Ogre*-like retrotransposons (Figure 4C and Figure S7D,E).

Similarly, we detected multiple VaccSat7-like units within two non-autonomous *Harbinger*-type DNA transposon families which are flanked by 3 bp TSDs, mostly consisting of a TAA or TTA trinucleotide. Copies of *HARB-N1_VCo* from *V. corymbosum* and *HARB-N7_VMa* from *V. macrocarpon* contain 3–5 units of VaccSat7 in tandem (Figure 4D). The VaccSat7 similarity corresponds to the sequence near the 3′ terminus of the *Harbinger* transposons (Figure 4D and Figure S7F,G).

*HARB-N11_VMa* and *HARB-N12_VMa* were distant relatives of the above-mentioned *Harbinger* transposons found in the genome of *V. macrocarpon.* These two transposon families do not contain VaccSat7-like repeat units in tandem. However, the 3′ termini of these families show sequence similarity to the termini of *HARB-N1_VCo* and *HARB-N7_VMa.* This suggests that VaccSat7 was generated by the multiplication of terminal part of a *Harbinger* DNA transposon (Figure 4D and Figure S7F,G).

### 3.4. Pattern of Distribution and Number of Monomers of Each Satellite Repeat on the Pseudochromosomes

Although genome assemblies are intrinsically error-prone and unsuitable for repeat quantification, analysis of chromosome scaffolds may still provide unique insights into the positioning of satellite DNA families along the pseudochromosomes. Here, we located VaccSat1, VaccSat2, VaccSat5, and VaccSat6 in most of the pseudochromosomes, while VaccSat3 and VaccSat7 covered the full-length of all of the pseudochromosomes of the tetraploid *V. corymbosum* cultivar “Draper.”

**Figure 5:**
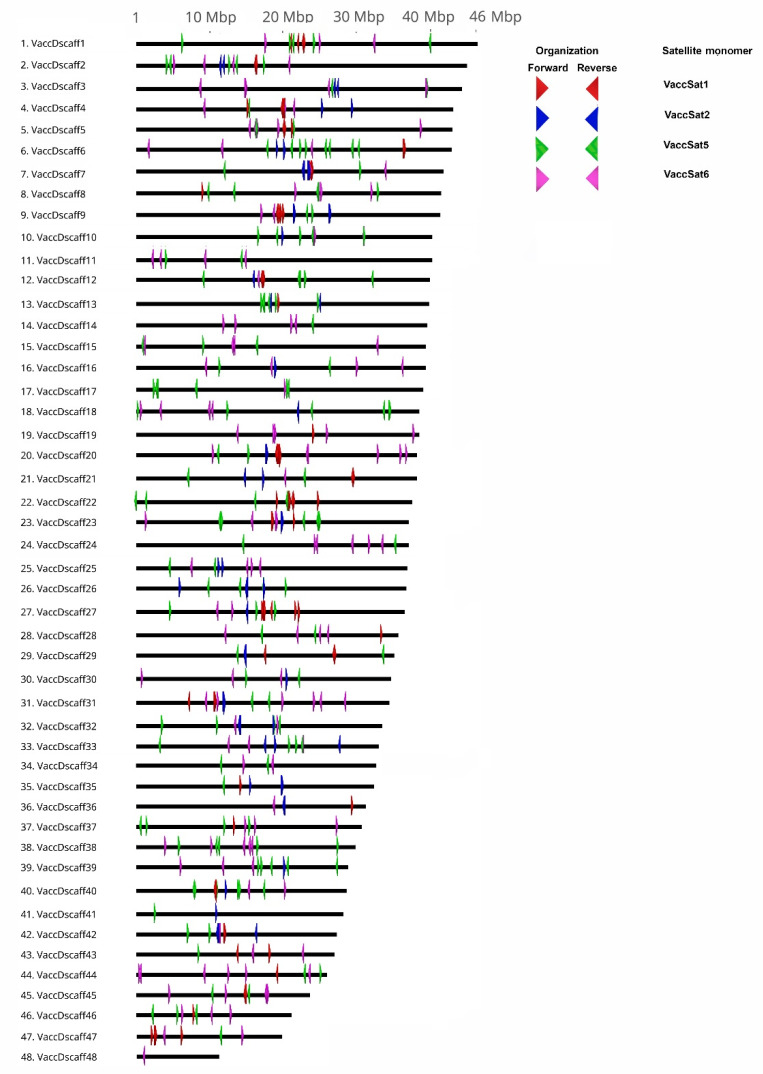
Distribution of the tandemly organized satellite families VaccSat1, VaccSat2, VaccSat5, and VaccSat6 along the 48 *V. corymbosum* pseudochromosomes. The individual VaccSat families are distinguished by arrowhead color, with red = VaccSat1, blue = VaccSat2, green = VaccSat5, and purple = VaccSat6. Forward and reverse oriented arrows indicate the orientation of monomers and arrays.

More specifically, VaccSat1 and VaccSat2 were detected in 26 and 28 out of 48 pseudochromosomes, respectively. Both satellite repeats were found to be located close to each other in the central region of the pseudochromosomes (Figure 5). In contrast, VaccSat5 and VaccSat6 have a tandem organization in comparatively smaller arrays and multiple sites on the chromosomes, identified along all except two pseudochromosomes (Figure 5). In addition, these two satellite repeats accumulate in the central and distal pseudochromosome regions. The dispersed satellite repeats VaccSat3 and VaccSat7 cover the complete length of all pseudochromosomes with a relatively even coverage (Figure S8A–C).

## 4. Discussion

The comparative analysis of repeat distributions may offer insights into genome evolutionary patterns in a taxon and ploidy-dependent manner. This strategy was already applied to many plant taxa such as *β* [31], *Dendrobium* [68], *Camellia* [4], the Fabeae [69], or *Linum* [70]. Here, we characterized the sequence diversity, periodicity and homogeneity of six tandemly organized repeats in four *Vaccinium* species (*V. corymbosum* cultivar “Jubilee,” *V. arctostaphylos*, *V. myrtillus*, *V. uliginosum*), and compared these to the reference species *V. corymbosum* strain “W850” and *V. macrocarpon* cultivar “Ben Lear.” The relative phylogenetic relationship among all the studied species was recalculated from the concatenated published sequence data of *Vaccinium*-specific chloroplast maturase K gene (*matK* gene) and nuclear ribosomal internal transcribed spacer gene (*nrITS* gene) by Powell and Kron, (2002) [44]. It was found that *V. myrtillus* and *V. macrocarpon* were closely related and formed a sister clade, well separated from the other *Vaccinium* species (Figure S1).

Here, primers for satellite amplification were designed to reveal the maximum diversity within and among the species, followed by cloning of PCR fragments from different species. Such a strategy ensures the amplification of representative satellite repeats from different species [44]. Nevertheless, amplification of satellite DNA by PCR has also drawbacks, such as a potential amplification of chimeric amplicons. As PCR products of tandem repeats may also act as primers in the next cycle, we cannot confidently exclude the presence of artificial sequences in our setup. Therefore, apart from the satellite DNA variability in the analyzed genomes analyzed, an influence of the DNA quality and amount, primer binding efficiency, and annealing temperature on VaccSat amplification differences has to be taken into consideration. We have therefore adjusted our PCR conditions to increase the specificity of the amplified products. As PCR is quick, cheap, and the most available laboratory method to differentiate between closely related genomes, our analysis may demonstrate how to offers insights into the monomer heterogeneity of satDNA with limited resources. For *Vaccinium*, we hope to inform future analyses of the genus and its repeats.

Our investigations indicated similar that VaccSat2, VaccSat3, and VaccSat7 sequences have similar amplification patterns in all *Vaccinium* species studied. In contrast, VaccSat1, VaccSat5, and VaccSat6 show some degree of a species-specific divergence of satellite monomers. Species-specific divergence of satellite repeat arrays is a well-known phenomenon and has been studied in species of many genera, such as *Solanum* [71], *β* [72], *Vicia* [8,73], and *Camellia* [4], respectively, and many other plant and animal genomes [74,75]. Rapid satellite DNA diversification after speciation, often accompanied by replacement or elimination of the common satellite repeat progenitor is generally explained by the mechanisms of molecular drive [29,76]. Although hard to pinpoint, we assume unequal chromosome exchange, extrachromosomal circular DNA and rolling circle replication, to be responsible for the species-specific divergence of satellite DNA [6,77,78].

The satellite repeat monomer lengths are largely consistent across the *Vaccinium* species tested, ranging between 36–240 bp for the six VaccSat families. It has been postulated that monomer sizes between 160–180 bp and 320–360 bp could facilitate DNA wrapping and phasing around the nucleosomes, recognized as important for DNA packaging or centromere function in many species [1,30,74,79]. Consistently, for VaccSat1 and VaccSat2 a central position has been observed along the *V. corymbosum* pseudochromosomes, while for VaccSat5, and VaccSat6 association with multiple locations along the pseudochromosomes are found. As the 48 *V. corymbosum* chromosomes are mainly metacentric and submetacentric [80], the VaccSat1 and VaccSat2 satellite positions may overlap with the centromere. Nevertheless, this positional information may only serve as a first approximation, as genome assemblies are prone to error, and especially repeats are often misassembled [81,82,83].

We identified AT-rich regions in all VaccSat satellites, with VaccSat1, VaccSat5 and VaccSat6 harboring overrepresentated AA/TT-rich regions (Table 2). AT-rich regions are presumably linked to the bending of the DNA molecules and differential DNA packaging in euchromatic and heterochromatic regions [74,75,77]. In contrast, VaccSat2 monomers harbor GC-rich regions (Table 2), potential targets for RNA-directed DNA methylation and epigenetic modifications [6,27,77]. The analyzed VaccSat7 monomers are highly heterogeneous due to the addition or deletion of “CAAAAAA” and similar “CAAAA” motifs, assumed to be linked to the breakage-reunion cycle of satellite repeats [74,77,84].

Although PCR of repetitive regions may lead to chimeric, artificial multimers, we want to cautiously report our findings regarding the HOR structure of *Vaccinium* satDNA: VaccSat1, VaccSat2, VaccSat5, and VaccSat6 likely exhibit HOR arrangements and are constituted of two different subunits. While one of the two HOR subunits had a relatively constant sequence length and composition throughout all species, the other HOR subunit was more diverse. Therefore, in *Vaccinium*, the second HOR subunit may be more significant as a potential marker for species identification and complexity. The formation of satellite subunits, the organization of monomers in HOR structures, and the subsequent multimer formation have been described in many different plant species, e.g., in the family of Fabaceae [73], Amaranthaceae [31,34,85], Theaceae [4], Iridaceae [86], or sweet grasses [30]. HOR structures of satellite repeats are directly linked with the formation of new tandem repeats of larger monomer units and the diversification of satellite repeats [6] which may also apply to *Vaccinium*.

VaccSat2 VaccSat3, and VaccSat7 are homogeneous and show high sequence similarity among and across the species, with only random deletions of certain sequence motifs. Genome-specific divergence was not detected for these three satellite families in different *Vaccinium* species. In contrast, VaccSat1, VaccSat5, and VaccSat6 are more heterogeneous across the species. Moreover, VaccSat1, VaccSat5, and VaccSat6 comprised species-specific sequence variation. High genome-wide sequence homogeneity is thought to be the result of concerted evolution, with more homogeneous sequences on the intra-species level, and more heterogeneous sequences on the inter-species level [7,87]. Garrido-Ramos (2017) [6] explained that sequence homogenization is directly linked to several factors, such as reproduction, environment, chromosomal localization, copy number or even functional constraints. For instance, different homogenization has been observed on the Y chromosomes of human [88] and *Rumex acetosa* [89]. As Y-linked satDNA homogenizes in a chromosome-specific manner, Y chromosomes cannot recombine anymore during meiosis. Contrarily, subtelomeric satellite repeats often show high sequence diversity, because those particular locations of the chromosomes correspond to recombination hot spots [15,73,90,91]. Moreover, the highly heterogeneous nature could also be linked to comparatively young satellite repeats with incomplete homogenization [73].

The complex satellite repeat organization observed here can be explained by two antagonistic evolutionary models, known as “concerted” and “satellite library” model (reviewed in references [6,29]). Whereas the concerted evolution model is generally used to explain the gradual accumulation of satellite repeat divergence with increasing species distances, the satellite library model also explains the often patchy distribution of the same satellite family in distantly related species. The mechanisms underlying the concerted evolution have been summarized by Dover (1982, 2002) coining the term “molecular drive”: the accumulation of mutations in the sequence motif is followed by homogenization of the mutated motif and fixation in the population [76,92]. All evolutionary steps are influenced by speciation, natural selection, genetic drift, and time. Complementing the concerted evolution model, it has been speculated for more than 40 years that a “satellite library” contained the total amount of satellite repeats present in an organism and could serve as source of satellite repeats for the future generation [6,8,93].

We detected multiple associations of transposable elements with satellite repeats, often as internal part of the transposons. On a technical level, this may impact the copy number estimation for the individual repeats or localization along the scaffolds, as differentiation between TE and satellites may be impaired. The presence of sequences similar to VaccSat1 and VaccSat6 in LTR retrotransposons may suggest that some of satellite sequences originated from parts of TEs or vice versa, some satellite repeats could have been randomly acquired by TEs. In addition, the absence of VaccSat1 in the genome of *V. macrocarpon* and the presence of TEs having the sequences similar to VaccSat1 in the same genome may also support the TEs’ function as a satellite library. The presence of repeated arrays of VaccSat3 and VaccSat7 in DNA transposons may also indicate a potential satellite dispersal in the genome by TEs. This may in turn affect the homogenization of the satellite repeats. Hence, different chromosome of the same species and even closely related species might show different satellite repeat profiles. Similar interspersion of satellite sequences and TEs have been observed also in other plant and animal genomes, such as in *Silene latifolia* [94], *Pisum sativum* [25], *Lathyrus sativus* [95], and *Drosophila melanogaster* [96], showing the generality of this phenomenon [24].

Moreover, VaccSat1 and VaccSat2 occur each in about half of the 48 highbush blueberry pseudochromosomes only and are either absent or reduced in the other half. These distribution patterns may indicate a subgenome-specific dispersal, which may reflect the repeat profiles of the two parental species. This could be a further indication towards an allotetraploid nature of highbush blueberry [48], with ongoing inter-subgenome dispersal of satDNA.

As our analyses contribute to reveal the evolutionary mechanisms that lead to rapid satellite divergence and proliferation, our results may help to establish the phylogenetic relationship among closely related species [97] as well as their use as molecular markers for species identification or characterization [8].

## 5. Conclusions

We identified and characterized six satellite DNAs in four *Vaccinium* species, provided primer pairs for PCR, and analyzed their organization, sequence heterogeneity, similarity with TEs, and distribution, providing insight into *Vaccinium* genome dynamics. The satellite DNA homogenization and amplification patterns were highly diverse among the studied species. As new repeat-based methods are put forward to harness the phylogenetic signal of related species, our results may open the way toward understanding the complicated species relationships in *Vaccinium*.

## Figures and Tables

**Table 1:** Genus, section, origin, location, chromosome number, and reference of the studied *Vaccinium* species. NOHU = Nigde Omer Halisdemir University, KM = Kaçkar Mountains, Rize, Turkey.

Species	Section *	General Information of Specimens					Reference
		Place of Collection	Latitude	Longitude	Elevation [m above Sea Level]	Chromosome Number	
*Vaccinium corymbosum* L. (cultivar ‘Jubilee’)	*Cyanococcus*	Turkey (NOHU)	37.563200	34.372500	1,300	48	Trehane, 2004 [36]
*V. corymbosum* L. (cultivar ‘Misty’)	*Cyanococcus*	Turkey (NOHU)	37.940462	37.940462	1,300	48	Trehane, 2004 [36]
*Vaccinium corymbosum* strain W8520	*Cyanococcus*	-	-	-	-	24	Sultana et al., 2017 [51]
*V. corymbosum* L. (cultivar ‘Draper’)	*Cyanococcus*	-	-	-	-	48	Colle et al., 2019 [48]
*Vaccinium arctostaphylos* L. (round shaped fruit)	*Hemimyrtillus*	Turkey (KM)	40.840298	41.102927	2641	48	Sultana et al., 2019 [50]
*V. arctostaphylos* L. (elongated shaped fruit)	*Hemimyrtillus*	Turkey (KM)	40.843791	41.081443	2779	48	Sultana et al., 2019 [50]
*Vaccinium myrtillus* L.	*Myrtillus*	Turkey (KM)	40.952462	41.101182	1251	24	Sultana et al., 2019 [50]
*Vaccinium uliginosum* L.	*Vaccinium*	Turkey (KM)	40.952461	41.101182	1251	24	Sultana et al., 2019 [50]
*Vaccinium macrocarpon* cultivar ‘Ben Lear’	*Oxycococus*	-	-	-	-	24	Sultana et al., 2017 [51]

* Section or subgeneric classification was performed according to Van der Kloet and Dickinson, 2009 [40].

**Table 2:** Primer pairs for satellite amplification.

Satellite Repeat	Forward Primer (5′–3′)	Reverse Primer (5′–3′)	Product Size (bp)	Annealing Temperature Used (°C)
VaccSat1/VaccSat4	ATTTAAAATGATTTTGTCGC	GCAAATAATAATGGTATTTAGC	119	48.9
VaccSat2	GTACGGGCTACTGACCAC	TATCGCTCAAACAACAAGTGG	198	56.8
VaccSat3	ATTTGACATTGTTGGCTTGC	GATCTCAATTAGTAGTTTAATTTGGTG	111	55.2
VaccSat5	ATTAAATCCATTTAAATCATTTTCTG	GATTTAAATGGATTTAATTAAAAATCC	55	51.3
VaccSat6	CTGACGGATTTTAAAAACGATG	TCCGTCAGGTATTATTATGATTTTC	54	54.4
VaccSat7	CAAGTTAGTTTTTTTGCAAAAC	GTTTTGCAAAAAAACTAACTTG	49–70	51.9

**Table 3:** Characteristics of *Vaccinium* satellite repeats as detected by read cluster analysis of diploid *V. corymbosum* strain W8520 [46].

Satellite Repeat	Monomer Length (bp)	G/C Content (%)	Genome Proportion (%) ^a^	Copy Number ^b^
VaccSat1	146–147	18.4	0.999	31,439
VaccSat2	238	40.9	0.124	2436
VaccSat3	154	21.1	0.36	10,987
VaccSat4	101	17.4	0.050	2326
VaccSat5	36–38	19.9	0.015	1905
VaccSat6	49	22.4	0.015	1438
VaccSat7	49–70	31.4	0.069	4632 to 6618

^a^ from RepeatExplorer output (Sultana et al. 2017 [46]); ^b^ copy number = genome size * genome proportion/monomer length.

**Table 4:** Number of satellite clones, monomer sequences and similarity percentage of extracted full-length monomer sequences with consensus monomer sequences in different species of *Vaccinium*, Empty (-) = No consistent multimeric PCR amplification and hence no plasmid clone.

Satellite Family	Species	Number of Clones	Number of Monomers	Identity to Monomer Consensus [%]
VaccSat1/VaccSat4	*V. corymbosum*	8	16	78–92
	*V. arctostaphylos*	4	11	68–92
	*V. myrtillus*	2	4	67–86
	*V. uliginosum*	7	10	83–97
VaccSat2	*V. corymbosum*	2	4	86–88
	*V. arctostaphylos*	4	7	82–90
	*V. myrtillus*	4	11	83–90
	*V. uliginosum*	4	6	71–79
VaccSat3	*V. corymbosum*	3	3	88–90
	*V. arctostaphylos*	4	4	84–90
	*V. myrtillus*	4	11	65–91
	*V. uliginosum*	4	10	65–89
VaccSat5	*V. corymbosum*	1	1	-
	*V. arctostaphylos*	4	20	88–96
	*V. myrtillus*	2	5	96–97
	*V. uliginosum*	-	-	-
VaccSat6	*V. corymbosum*	1	1	-
	*V. arctostaphylos*	4	14	89–90
	*V. myrtillus*	5	19	81–88
	*V. uliginosum*	-	-	-
VaccSat7	*V. corymbosum*	-	-	-
	*V. arctostaphylos*	5	13	66–75
	*V. myrtillus*	4	10	69–78
	*V. uliginosum*	-	-	-
**Total**		**76**	**180**	

## Supplementary Materials

The following are available online at https://www.mdpi.com/2073-4425/11/5/527/s1, Figure S1: Phylogenetic relationship among the analyzed *Vaccinium* species, Figure S2: Satellite repeat structure and primer binding sites, Figure S3: Dotplot analysis of multimeric satellite repeats, Figure S4: Multiple sequence alignment of full-length monomer sequences from different *Vaccinium* species of six satellite repeats, Figure S5: Analysis of subunit structure of VaccSat1 in different *Vaccinium* species, Figure S6: Analysis of subunit structure of VaccSat2 in different *Vaccinium* species, Figure S7: Similarities between satellite repeats and TEs, Figure S8: Distribution of dispersedly distributed satellite repeats along the pseudochromosomes of tetraploid *Vaccinium corymbosum* genome, Table S1: Scoring of successful PCR amplification and clone overview, Table S2: Summary of the satellite repeat structure and organization in the genome of different species of *Vaccinium.* (HOR = higher order repeat structure of satellite monomer), Table S3: Effect of homogenous primer regions on the consensus full-length monomer identity.

## References

[1] Schmidt T., Heslop-Harrison J.S. (1998). Genomes, genes and junk: The large-scale organization of plant chromosomes. Trends Plant Sci..

[2] Heslop-Harrison J.S.P., Schwarzacher T. (2011). Organisation of the plant genome in chromosomes. Plant J..

[3] Kelly L.J., Renny-Byfield S., Pellicer J., Macas J., Novák P., Neumann P., Lysak M.A., Day P.D., Berger M., Fay M.F. (2015). Analysis of the giant genomes of *Fritillaria* (*Liliaceae*) indicates that a lack of DNA removal characterizes extreme expansions in genome size. New Phytol..

[4] Heitkam T., Petrasch S., Zakrzewski F., Kögler A., Wenke T., Wanke S., Schmidt T. (2015). Next-generation sequencing reveals differentially amplified tandem repeats as a major genome component of Northern Europe’s oldest *Camellia japonica*. Chromosome Res..

[5] Kirov I.V., Kiseleva A.V., Van Laere K., Van Roy N., Khrustaleva L.I. (2017). Tandem repeats of *Allium fistulosum* associated with major chromosomal landmarks. Mol. Genet. Genom..

[6] Garrido-Ramos M. (2017). Satellite DNA: An evolving topic. Genes.

[7] Garrido-Ramos M.A. (2015). Satellite DNA in plants: More than just rubbish. Cytogenet. Genome Res..

[8] Ávila Robledillo L., Koblížková A., Novák P., Böttinger K., Vrbová I., Neumann P., Schubert I., Macas J. (2018). Satellite DNA in *Vicia faba* is characterized by remarkable diversity in its sequence composition, association with centromeres, and replication timing. Sci. Rep..

[9] Iwata A., Tek A.L., Richard M.M.S., Abernathy B., Fonsêca A., Schmutz J., Chen N.W.G., Thareau V., Magdelenat G., Li Y. (2013). Identification and characterization of functional centromeres of the common bean. Plant J..

[10] Zhang H., Koblížková A., Wang K., Gong Z., Oliveira L., Torres G.A., Wu Y., Zhang W., Novák P., Buell C.R. (2014). Boom-bust turnovers of megabase-sized centromeric DNA in *Solanum* species: Rapid evolution of DNA sequences associated with centromeres. Plant Cell.

[11] He Q., Cai Z., Hu T., Liu H., Bao C., Mao W., Jin W. (2015). Repetitive sequence analysis and karyotyping reveals centromere-associated DNA sequences in radish (*Raphanus sativus* L.). BMC Plant Biol..

[12] Jagannathan M., Cummings R., Yamashita Y.M. (2018). A conserved function for pericentromeric satellite DNA. Elife.

[13] Mizuno H., Wu J., Kanamori H., Fujisawa M., Namiki N., Saji S., Katagiri S., Katayose Y., Sasaki T., Matsumoto T. (2006). Sequencing and characterization of telomere and subtelomere regions on rice chromosomes 1S, 2S, 2L, 6L, 7S, 7L and 8S. Plant J..

[14] Churikov D., Price C.M. (2008). Telomeric and Subtelomeric Repeat Sequences. Encyclopedia of Life Sciences.

[15] Torres G.A., Gong Z., Iovene M., Hirsch C.D., Buell C.R., Bryan G.J., Novák P., Macas J., Jiang J. (2011). Organization and evolution of subtelomeric satellite repeats in the potato genome. G3 Genes Genomes Genetics.

[16] Shapiro J.A., von Sternberg R. (2005). Why repetitive DNA is essential to genome function. Biol. Rev..

[17] Ugarkovic D. (2005). Functional elements residing within satellite DNAs. EMBO Rep..

[18] Steflova P., Tokan V., Vogel I., Lexa M., Macas J., Novak P., Hobza R., Vyskot B., Kejnovsky E. (2013). Contrasting patterns of transposable element and satellite distribution on sex chromosomes (XY1Y2) in the dioecious plant *Rumex acetosa*. Genome Biol. Evol..

[19] Puterova J., Razumova O., Martinek T., Alexandrov O., Divashuk M., Kubat Z., Hobza R., Karlov G., Kejnovsky E. (2017). Satellite DNA and transposable elements in seabuckthorn (*Hippophae rhamnoides*), a dioecious plant with small Y and large X chromosomes. Genome Biol. Evol..

[20] Neumann P., Navrátilová A., Koblížková A., Kejnovský E., Hřibová E., Hobza R., Widmer A., Doležel J., Macas J. (2011). Plant centromeric retrotransposons: A structural and cytogenetic perspective. Mob. DNA.

[21] Wendel J.F., Jackson S.A., Meyers B.C., Wing R.A. (2016). Evolution of plant genome architecture. Genome Biol..

[22] Chuong E.B., Elde N.C., Feschotte C. (2016). Regulatory activities of transposable elements: From conflicts to benefits. Nat. Rev. Genet..

[23] Pellicer J., Hidalgo O., Dodsworth S., Leitch I.J. (2018). Genome size diversity and its impact on the evolution of land plants. Genes.

[24] Meštrović N., Mravinac B., Pavlek M., Vojvoda-Zeljko T., Šatović E., Plohl M. (2015). Structural and functional liaisons between transposable elements and satellite DNAs. Chromosome Res..

[25] Macas J., Koblížková A., Navrátilová A., Neumann P. (2009). Hypervariable 3′ UTR region of plant LTR-retrotransposons as a source of novel satellite repeats. Gene.

[26] Stupar R.M., Song J., Tek A.L., Cheng Z., Dong F., Jiang J. (2002). Highly condensed potato pericentromeric heterochromatin contains rDNA-related tandem repeats. Genetics.

[27] Mehrotra S., Goyal V. (2014). Repetitive sequences in plant nuclear DNA: Types, distribution, evolution and function. Genom. Proteom. Bioinform..

[28] Del Bosque M.E.Q., López-Flores I., Suárez-Santiago V.N., Garrido-Ramos M.A. (2014). Satellite-DNA diversification and the evolution of major lineages in *Cardueae* (*Carduoideae Asteraceae*). J. Plant Res..

[29] Plohl M., Meštrovic N., Mravinac B., Garrido-Ramos M.A. (2012). Satellite DNA Evolution. Repetitive DNA Genome Dynamic.

[30] Yang X., Zhao H., Zhang T., Zeng Z., Zhang P., Zhu B., Han Y., Braz G.T., Casler M.D., Schmutz J. (2018). Amplification and adaptation of centromeric repeats in polyploid switchgrass species. New Phytol..

[31] Menzel G., Dechyeva D., Wenke T., Holtgräwe D., Weisshaar B., Schmidt T. (2008). Diversity of a complex centromeric satellite and molecular characterization of dispersed sequence families in sugar beet (*Beta vulgaris*). Ann. Bot..

[32] Cechova M., Harris R.S., Tomaszkiewicz M., Arbeithuber B., Chiaromonte F., Makova K.D. (2019). High satellite repeat turnover in great apes studied with short- and long-read technologies. Mol. Biol. Evol..

[33] Lunerová J., Herklotz V., Laudien M., Vozárová R., Groth M., Kovařík A., Ritz C.M. (2020). Asymmetrical canina meiosis is accompanied by the expansion of a pericentromeric satellite in non-recombining univalent chromosomes in the genus *Rosa*. Ann. Bot..

[34] Heitkam T., Weber B., Walter I., Liedtke S., Ost C., Schmidt T. (2020). Satellite DNA landscapes after allotetraploidisation of quinoa (*Chenopodium quinoa*) reveal unique A and B subgenomes. Plant J..

[35] Belyayev A., Josefiová J., Jandová M., Kalendar R., Krak K., Mandák B. (2019). Natural history of a satellite DNA family: From the ancestral genome component to species-specific sequences, concerted and non-concerted evolution. Int. J. Mol. Sci..

[36] Trehane J. (2004). Blueberries, Cranberries, and other Vacciniums. Plant Collector Guide.

[37] Hancock J.F., Lyrene P., Finn C.E., Vorsa N., Lobos G.A., Hancock J.F. (2008). Blueberries and Cranberries. Temperate Fruit Crop Breeding: Germplasm to Genomics.

[38] Vander Kloet S.P. (1988). The genus Vaccinium in North America.

[39] Ehlenfeldt M.K., Ballington J.R. (2012). *Vaccinium* species of section *Hemimyrtillus*: Their value to cultivated blueberry and approaches to utilization. Botany.

[40] Vander Kloet S.P., Dickinson T.A. (2009). A subgeneric classification of the genus *Vaccinium* and the metamorphosis of *V*. section *Bracteata Nakai*: More terrestrial and less epiphytic in habit, more continental and less insular in distribution. J. Plant Res..

[41] Mudd A.B., White E.J., Bolloskis M.P., Kapur N.P., Everhart K.W., Lin Y.C., Bussler W.W., Reid R.W., Brown R.H. (2013). Students’ perspective on genomics: From sample to sequence using the case study of blueberry. Front. Genet..

[42] Ballington J.R. (2001). Collection, utilization, and preservation of genetic resources in Vaccinium. HortScience.

[43] Vicient C.M., Casacuberta J.M. (2017). Impact of transposable elements on polyploid plant genomes. Ann. Bot..

[44] Powell E.A., Kron K.A. (2002). Hawaiian blueberries and their relatives—A phylogenetic analysis of *Vaccinium* sections *Macropelma*, *Myrtillus*, and *Hemimyrtillus* (*Ericaceae*). Syst. Bot..

[45] Rowland L.J., Bell D.J., Alkharouf N., Bassil N.V., Drummond F.A., Beers L., Buck E.J., Finn C.E., Graham J., McCallum S. (2012). Generating genomic tools for blueberry improvement. Int. J. Fruit Sci..

[46] Zdepski A., Debnath S.C., Howell A., Polashock J., Oudemans P., Vorsa N., Michael T.P., Folta K., Kole C. (2011). Cranberry. Genetics, Genomics and Breeding of Berries.

[47] Costich D.E., Ortiz R., Meagher T.R., Bruederle L.P., Vorsa N. (1993). Determination of ploidy level and nuclear DNA content in blueberry by flow cytometry. Theor. Appl. Genet..

[48] Colle M., Leisner C.P., Wai C.M., Ou S., Bird K.A., Wang J., Wisecaver J.H., Yocca A.E., Alger E.I., Tang H. (2019). Haplotype-phased genome and evolution of phytonutrient pathways of tetraploid blueberry. Gigascience.

[49] Hummer K.E., Bassil N.V., Rodríquez Armenta H.P., Olmstead J.W. (2015). *Vaccinium* species ploidy assessment. Acta Hortic..

[50] Sultana N., Pascual-Díaz J.P., Gers A., Ilga K., Serçe S., Vitales D., Garcia S. (2019). Contribution to the knowledge of genome size evolution in edible blueberries (genus *Vaccinium*). J. Berry Res..

[51] Sultana N., Menzel G., Heitkam T., Schmidt T., Serce S. (2017). Comparative analysis of repetitive sequences reveals genome differences between two common cultivated *Vaccinium* Species (*V. corymbosum* and *V. macrocarpon*). J. Mol. Biol. Biotech..

[52] Fajardo D., Senalik D., Ames M., Zhu H., Steffan S.A., Harbut R., Polashock J., Vorsa N., Gillespie E., Kron K. (2012). Complete plastid genome sequence of *Vaccinium macrocarpon*: Structure, gene content, and rearrangements revealed by next generation sequencing. Tree Genet. Genomes.

[53] Polashock J., Zelzion E., Fajardo D., Zalapa J., Georgi L., Bhattacharya D., Vorsa N. (2014). The American cranberry: First insights into the whole genome of a species adapted to bog habitat. BMC Plant Biol..

[54] Gupta V., Estrada A.D., Blakley I., Reid R., Patel K., Meyer M.D., Andersen S.U., Brown A.F., Lila M.A., Loraine A.E. (2015). RNA-Seq analysis and annotation of a draft blueberry genome assembly identifies candidate genes involved in fruit ripening, biosynthesis of bioactive compounds, and stage-specific alternative splicing. Gigascience.

[55] Bian Y., Ballington J., Raja A., Brouwer C., Reid R., Burke M., Wang X., Rowland L.J., Bassil N., Brown A. (2014). Patterns of simple sequence repeats in cultivated blueberries (*Vaccinium* section *Cyanococcus* spp.) and their use in revealing genetic diversity and population structure. Mol. Breed..

[56] Li L., Zhang H., Liu Z., Cui X., Zhang T., Li Y., Zhang L. (2016). Comparative transcriptome sequencing and de novo analysis of *Vaccinium corymbosum* during fruit and color development. BMC Plant Biol..

[57] Novak P., Neumann P., Pech J., Steinhaisl J., Macas J. (2013). RepeatExplorer: A Galaxy-based web server for genome-wide characterization of eukaryotic repetitive elements from next-generation sequence reads. Bioinformatics.

[58] Kearse M., Moir R., Wilson A., Stones-Havas S., Cheung M., Sturrock S., Buxton S., Cooper A., Markowitz S., Duran C. (2012). Geneious Basic: An integrated and extendable desktop software platform for the organization and analysis of sequence data. Bioinformatics.

[59] Katoh K., Standley D.M. (2013). MAFFT multiple sequence alignment software version 7: Improvements in performance and usability. Mol. Biol. Evol..

[60] Price M.N., Dehal P.S., Arkin A.P. (2010). FastTree 2—approximately maximum-likelihood trees for large alignments. PLoS ONE.

[61] Benson G. (1999). Tandem repeats finder: A program to analyze DNA sequences. Nucleic Acids Res..

[62] Rice P., Longden I., Bleasby A. (2000). EMBOSS: The the European molecular biology open software suite. Trends Genet..

[63] Seibt K.M., Schmidt T., Heitkam T. (2018). FlexiDot: Highly customizable, ambiguity-aware dotplots for visual sequence analyses. Bioinformatics.

[64] Kohany O., Gentles A.J., Hankus L., Jurka J. (2006). Annotation, submission and screening of repetitive elements in Repbase: Repbase Submitter and Censor. BMC Bioinform..

[65] Blastclust. Ftp://ftp.ncbi.nih.gov/blast/documents/blastclust.html/.

[66] Kapitonov V.V., Jurka J. (2008). A universal classification of eukaryotic transposable elements implemented in Repbase. Nat. Rev. Gen..

[67] Bao W., Kojima K.K., Kohany O. (2015). Repbase Update, a database of repetitive elements in eukaryotic genomes. Mob. DNA.

[68] Begum R., Alam S.S., Menzel G., Schmidt T. (2009). Comparative molecular cytogenetics of major repetitive sequence families of three *Dendrobium* species (*Orchidaceae*) from Bangladesh. Ann. Bot..

[69] Macas J., Novák P., Pellicer J., Čížková J., Koblížková A., Neumann P., Fuková I., Doležel J., Kelly L.J., Leitch I.J. (2015). In depth characterization of repetitive DNA in 23 plant genomes reveals sources of genome size variation in the legume tribe Fabeae. PLoS ONE.

[70] Bolsheva N.L., Melnikova N.V., Kirov I.V., Dmitriev A.A., Krasnov G.S., Amosova A.V., Samatadze T.E., Yurkevich O.Y., Zoshchuk S.A., Kudryavtseva A.V. (2019). Characterization of repeated DNA sequences in genomes of blue-flowered flax. BMC Evol. Biol..

[71] Tek A.L., Song J., Macas J., Jiang J. (2005). Sobo, a recently amplified satellite repeat of potato, and its implications for the origin of tandemly repeated sequences. Genetics.

[72] Zakrzewski F., Weber B., Schmidt T., Jiang J., Birchler J.A. (2013). A Molecular Cytogenetic Analysis of the Structure, Evolution, and Epigenetic Modifications of Major DNA Sequences in Centromeres of *Beta* Species. Plant Centromere Biology.

[73] Macas J., Navrátilová A., Koblízková A. (2006). Sequence homogenization and chromosomal localization of VicTR-B satellites differ between closely related *Vicia* species. Chromosoma.

[74] Sharma S., Raina S.N. (2005). Organization and evolution of highly repeated satellite DNA sequences in plant chromosomes. Cytogenet. Genome Res..

[75] Melters D.P., Bradnam K.R., Young H.A., Telis N., May M.R., Ruby J., Sebra R., Peluso P., Eid J., Rank D. (2013). Comparative analysis of tandem repeats from hundreds of species reveals unique insights into centromere evolution. Genome Biol..

[76] Dover G. (1982). Molecular drive: A cohesive mode of species evolution. Nature.

[77] Macas J., Mészáros T., Nouzová M. (2002). PlantSat: A specialized database for plant satellite repeats. Bioinformatics.

[78] Cohen S., Houben A., Segal D. (2008). Extrachromosomal circular DNA derived from tandemly repeated genomic sequences in plants. Plant J..

[79] Heslop-Harrison J.S.P., Schwarzacher T. (2013). Nucleosomes and centromeric DNA packaging. Proc. Natl. Acad. Sci. USA.

[80] Qu L., Hancock J.F., Whallon J.H. (1998). Evolution in an autopolyploid group displaying predominantly bivalent pairing at meiosis: Genomic similarity of diploid *Vaccinium darrowi* and autotetraploid *V. corymbosum* (*Ericaceae*). Am. J. Bot..

[81] Jain M., Olsen H.E., Turner D.J., Stoddart D., Bulazel K.V., Paten B., Haussler D., Willard H.F., Akeson M., Miga K.H. (2018). Linear assembly of a human centromere on the Y chromosome. Nat. Biotechnol..

[82] Miga K. (2015). Completing the human genome: The progress and challenge of satellite DNA assembly. Chromosome Res..

[83] Peona V., Weissensteiner M.H., Suh A. (2018). How complete are “complete” genome assemblies?—An avian perspective. Mol. Ecol. Resour..

[84] Mlinarec J., Chester M., Siljak-Yakovlev S., Papes D., Leitch A.R., Besendorfer V. (2009). Molecular structure and chromosome distribution of three repetitive DNA families in *Anemone hortensis* L. (*Ranunculaceae*). Chromosome Res..

[85] Zakrzewski F., Wenke T., Weisshaar B., Schmidt T., Holtgräwe D. (2010). Analysis of a c0t-1 library enables the targeted identification of minisatellite and satellite families in *Beta vulgaris*. BMC Plant Biol..

[86] Schmidt T., Heitkam T., Liedtke S., Schubert V., Menzel G. (2019). Adding color to a century-old enigma: Multi-color chromosome identification unravels the autotriploid nature of saffron (*Crocus sativus*) as a hybrid of wild *Crocus cartwrightianus* cytotypes. New Phytol..

[87] Plohl M. (2010). Those mysterious sequences of satellite DNAs. Period. Biol..

[88] Skaletsky H., Kuroda-Kawaguchi T., Minx P.J., Cordum H.S., Hillier L., Brown L.G., Repping S., Pyntikova T., Ali J., Bieri T. (2003). The male-specific region of the human Y chromosome is a mosaic of discrete sequence classes. Nature.

[89] Mariotti B., Manzano S., Kejnovský E., Vyskot B., Jamilena M. (2009). Accumulation of Y-specific satellite DNAs during the evolution of *Rumex acetosa* sex chromosomes. Mol. Genet. Genom..

[90] Richard M.M.S., Chen N.W.G., Thareau V., Pflieger S., Blanchet S., Pedrosa-Harand A., Iwata A., Chavarro C., Jackson S.A., Geffroy V.T. (2013). The subtelomeric khipu satellite repeat from *Phaseolus vulgaris*: Lessons learned from the genome analysis of the Andean genotype G19833. Front. Plant Sci..

[91] Koukalova B., Moraes A.P., Renny-Byfield S., Matyasek R., Leitch A.R., Kovarik A. (2010). Fall and rise of satellite repeats in allopolyploids of *Nicotiana* over c. 5 million years. New Phytol..

[92] Dover G. (2002). Molecular drive. Trends Genet..

[93] Fry K., Salser W. (1977). Nucleotide sequences of HS-alpha satellite DNA from kangaroo rat *Dipodomys ordii* and characterization of similar sequences in other rodents. Cell.

[94] Kejnovsky E., Kubat Z., Macas J., Hobza R., Mracek J., Vyskot B. (2006). Retand: A novel family of gypsy-like retrotransposons harboring an amplified tandem repeat. Mol. Genet. Genom..

[95] Vondrak T., Ávila Robledillo L., Novák P., Koblížková A., Neumann P., Macas J. (2020). Characterization of repeat arrays in ultra-long nanopore reads reveals frequent origin of satellite DNA from retrotransposon-derived tandem repeats. Plant J..

[96] McGurk M.P., Barbash D.A. (2018). Double insertion of transposable elements provides a substrate for the evolution of satellite DNA. Genome Res..

[97] Dodsworth S., Chase M.W., Kelly L.J., Leitch I.J., Macas J., Novak P., Piednoel M., Weiss-Schneeweiss H., Leitch A.R. (2014). Genomic repeat abundances contain phylogenetic signal. Syst. Biol..

